# Medical History of the Expedition to the Niger during the Years 1841-2, Comprising an Account of the Fever Which Led to Its Abrupt Termination

**Published:** 1843-07

**Authors:** 


					Art. XVII.
Medical History of the Expedition to the Niger during the years 1841-2;
comprising an account of the Fever which led to its abrupt termi-
nation.
By J. O. M'William, Surgeon of H. M. S. Albert, and
Senior Medical Officer of the Expedition. With Plates.?London,
1843. 8vo, pp. 296.
There are few of our readers but must remember the melancholy
impression made on the public mind by the disastrous result of the
expedition to the Niger, when this was made known in England
through the newspapers. And none who remember this can forget that
pathetic passage in the story, which represented the noble conduct of
the surgeon and the geologist of the expedition, when left alone, in
the far recesses of the Niger, amid their heroic companions all stricken
to death or to death-like helplessness by the fatal fever of the country.
In this trying conjuncture, when the salvation of all on board depended
on the speedy removal of the ship from her actual position, Dr. M'William
took the navigation on himself, steering with his own hand and pilot-
ing the vessel through all the intricacies of the river, while his com-
panion worked the engine below. There is something very affecting,
we had almost said sublime, in the picture thus presented to the imagi-
nation of these two solitary men of science assuming offices so foreign
260 Dr. M'William's Medical History [July*
to their past habits and knowledge, stripped of all exterior cogni-
zance of their class, standing as humble workmen at the helm and
furnace, toiling by day, watching by night, while the force of the stream
and the paddles was sweeping their ill-fated bark, freighted with their
dying or dead companions, through the manifold dangers of their un-
known course. The author of the volume before us was the clear-
headed and stout-hearted pilot who did this, the undoubted preserver
of the ship and her surviving crew ; and the slight and simple way in
which he speaks of his own exertions strikingly illustrates the old truth,
that the brave man is ever modest.
To visit with critical severity a book written under such circum-
stances would seem harsh in any case ; but we can conscientiously
say, that the volume before us stands in no need of such protecting in-
fluence. It is simply, clearly, and correctly written, and tells exactly
what the title-page and preface promise. It is truly a history, a narra-
tive of facts, unmixed with speculations; and an interesting history it is,
and one which must be referred to by all future explorers of Africa, and
all future inquirers into the nature of the fevers of that fatal country.
Though the field explored by Dr. M'William was ample, the living sub-
jects of his observation were comparatively few ; and on this account he
has wisely contented himself with giving the exact results witnessed,
without any attempt at generalising them or applying them to other
times and places.
The late period at which Dr. M'William's volume has reached us must
prevent our giving any other than a brief notice of its contents ; but we
earnestly recommend its perusal to all our readers. It is a model for the
composition of similar works.
The work is divided into three parts :
" The First comprises as much general description as is necessary to put the
reader in possession of those circumstances of position and climate which could
produce or modify disease.
"The Second contains an account of the fever as it occurred on board the
Albert, embracing its main features and treatment.
" In the Third Part will be found a few facts relating to the state of medicine
in the Ni^er, and to vaccination among the Blacks; a brief description of the
system of ventilation adopted in the ships, with some remarks on its employ-
ment on the coast and on the river; an abstract of the meteorological observa-
tions which were made after the plan recommended by the Royal Society; and
lastly, a brief account of the geology of the Niger
"The Expedition left England on the 12th of May, 1841, and entered the
Niger on the 13th of August. Three weeks from this period fever broke out
among the crews, and soon produced effects so disastrous that two of the three
steam-vessels composing the Expedition were obliged to return to the sea, and
the other was compelled to follow a few weeks after." (Preface, iii-iv.)
"The following is a list of officers, seamen, and marines, of men of colour, of
various nations, who joined the expedition in England; and of Kroomen and
liberated African boys entered on the Coast, who were on board H.M.S. Albert,
Wilberforce, Soudan, and Amelia, on their entrance into the Nun branch of the
river Niger:
1843.] of the Expedition to the Niger. 261
H. M. Ships
Albert ,
Amelia tender
Wilberforce
Soudan
Total of each class
">
'6 &
fao?
.5 a
l!
? Clj
" J
O) ^
sj
O be
14
S *
o
n
B
o
109
3T
106
51
53 63 29 145 25 110 23 158 303"
(p. 44.)
And the following Table gives at one view the total mortality from
the time the Expedition left England to its completion :
" Return of the Total Mortality stated under the respective Ships to which the Officers,
Seamen, Marines, Sf-c. belonged, from the time the Expedition left England to its
completion.
Average complement
Deaths from fever con-
tracted on the coast
in the river
Accidents
Other complaints
Albert, including
the Amelia, &c.
21
? cs
? co
3T
iX ?
146
WlLBERFORCE.
21
? a.
a a.
? cs
S?
36
2 4S
49
106
Soudan.
1
S e
c EL
ID o3
S cn
19
< M
O
cd
,2 5
2 *
53 s>
?a -o
21
51 303
2
13 42
1 6
3
14 53"
(P- 130.)
There appears to have been nothing peculiar in the disease of the
Niger : it was evidently the Bilious remittent fever of the older authors,
as will appear from the following extracts :
" The accession was seldom accompanied by very marked shivering, yet pre-
vious to the period of vascular excitement, the patient usually experienced a
262 Dr. M'William's Medical History
sensation of coldness, and for the sake of warmth would fain have exposed
himself to the rays of the sun. He would shortly express a wish to lie down,
and would complain somewhat suddenly of increase of headach or giddiness,
and intense heat of the skin, which had a dry parched feel, restlessness,
intolerable nausea, and difficult breathing. The dyspnea in several instances,
particularly in ray own case, was extremely distressing, and continued from one
to four hours, until relieved by spontaneous vomiting, or the occurrence of
diaphoresis. Headach was with some the prominent symptom during the hot
stage, and the feeling was described as that of a cord being tightly girded round
the temples. The thirst was very urgent; the tongue was foul in the centre,
moist, clean or reddish, and invariably marked by indentations on the edges.
The countenance was more or less flushed, the eye occasionally suffused and
always looked wild. Pulse rapid but small, frequently feeble; thirst urgent,
bowels constipated, and urine passed often and in small quantity. There was
in general tenderness of the epigastrium, sometimes acute, but often not dis-
coverable unless upon pressure.
"In some cases, coldness of the stomach was complained of some days before
death. A subsidence of febrile action in general followed in from three to six
hours, or at all events, the symptoms if continued beyond the latter period
became much mitigated. Diaphoresis came on, the thirst moderated, and the
signs of oppression in a great measure disappeared. The principal complaint
at this period was from the disagreeable odour of the perspiration, particularly in
those cases that subsequently proved fatal. I was not sensible of this peculiarity
in the smell of the perspiration in my own case, but I perceived it very dis-
tinctly in several others. The sweating continued until from eight to twelve
hours had been occupied by the whole paroxysm. The patient, although con-
siderably exhausted, expressed himself as free from all trouble, and the counte-
nance also indicated improvement. This seemingly favorable change did not
last .long, for the accession generally returned in from six to ten or twelve
hours. Occasionally the respite extended to twenty-four hours. In a few cases,
there was a treacherous interval of forty-eight hours, in the early period of the
disease; but these invariably assumed afterwards a low malignant type. The
fever in them seemed to have rested only to give strength for a fresh accession.
" The accessions did not seem to observe any law of periodicity. They came
on, disappeared, and returned at all hours of the day and night. The evening,
however, was a more common time of accession than any other ; in which case,
after the cold sensation had passed off, the paroxysm generally ran through its
stages in the course of the night, and had suffered a considerable remission by
the hour of breakfast (eight) the next morning.
" In a few instances the remissions were as complete as in the interval of
ague. These were, however, only exceptions to the general rule, for total
absence of fever was indeed of rare occurrence during the course of the disease.
" I cannot say that the influence of critical days was at all apparent, further
than if no material improvement was evident by the eighth or ninth day, the
prognosis was then most gloomy." (pp. 132-4.)
" When the disease was about to take a favorable turn, the remissions became
distinctly marked, and the intervals were lengthened. The countenance (the
best criterion) assumed a natural expression, a certain look of convalescence,
that one can only become acquainted with by experience and contrasting it with
that indicative of a fatal termination. The skin became moist, the thirst
diminished, the pulse was more voluminous and softer; the tongue gradually
lost its tremulousness, and could be more easily thrust out of the mouth; it
often continued a long time loaded, but the crust was less brown, and more
moist, and seemed to have lost its firm attachment to the organ : at this period
diarrhea was by no means uncommon, and also a copious flow of urine, which
latter was a very favorable symptom. A strong desire for food was expressed
by most of the patients who had advanced thus far, and I had more than once
cause to regret having gratified it." (p. 135.)
1843.] of the Expedition to the Niyer. 263
" Delirium was a very bad symptom in the fever of the Niger: of twenty-one
cases in which it occurred fourteen died, of whom one was drowned by eluding
his nurse, and jumping into the river." (p. 136.)
" Petechia or sudamina were not observed in any case
" Yellowness of the skin occurred in nineteen cases, thirteen of which were
fatal, and the average day of the appearance of this symptom was the ninth.
The yellow colour was first seen in the conjunctiva, and afterwards extended
over the face, arms, and the rest of the body. It was in general light, and did
not appear after death in any case in which it had not existed during life. The
faeces in these cases were generally of a bilious colour." (pp. 137-8.)
Only eight bodies of the dead could be examined by Dr. M'William :
the results, given in a concise and satisfactory form, present nothing
different from what has been recorded by preceding authorities. No
morbid peculiarity that could be fairly attributed to the fever, was found
in the head, thorax, or cavity of the abdomen. The mucous coat of the
stomach was invariably softened, occasionally marked with gorged
vessels and dark patches, and exhibiting in others small points of ulcera-
tion. The duodenum was affected in the same way as the stomach but
in a less degree. The ileum was softened towards its lower end, and
exhibited livid spots and also some ulcerations. Peyer's glands were
enlarged in three cases. The liver was large in two cases, anemious and
dry in two others. In three cases the gall-bladder was filled with tar-
like bile, and in a fourth this was mixed with blood. The spleen was
enlarged and softened in one case ; in a second, enlarged but firm. In
two cases the blood was found fluid several hours after death.
The author has an important section on the sequences of the fever,
which were chiefly chronic affections of the mucous membrane of the
bowels in the form of chronic dysentery, hemorrhage, and colic ; also
hepatitis. True intermittent fever was still more common.
"On board the Albert none of those who had fever in the Niger, and were
not at once sent to England, escaped intermittent. Five who suffered severely
were invalided at Ascension, nearly nine months after the vessel left the Niger.
In the Wilberforce nine cases of ague, following Niger fever, occurred during
the passage to Ascension. The severity of the intermittent did not always bear
a relation to the intensity of the primary remittent. Commander Fishbourne
was affected by the remittent in a comparatively mild form, but he was many
months afterwards as violently visited by intermittent as any one in the expedi-
tion. Nearly twelve months after recovery from remittent, and after about
eight months of freedom from intermittent, I was three days confined to bed,
with the latter complaint, during the passage to England, just as we were
getting into cold weather. It appears, therefore, that after remittent a person
continues long liable to intermittent, and further that remittent and intermittent
are produced by the same cause in different degrees of intensity." (pp. 154-5.)
The section on the causes of the fever is valuable, but throws no new
light on the subject, except to disprove the theory, promulgated some
time before the sailing of the expedition from England, that the existence
of free sulphuretted hydrogen gas in the waters of theNiger might account
for the fever. Dr. M'William shows in the most satisfactory manner
that no such gas is found in the Niger, and that what was detected in the
specimens sent to England, originated in the bottles themselves from de-
composition of their contents. The temperature was no doubt great,
" seldom under 84? on the lower deck," but a similar degree of heat
264 Dr. M'William's History of the Niger Expedition, [July,
exists elsewhere without producing fever. It also appears that this high
temperature was not combined with great moistness of the air, a combi-
nation so generally believed to be instrumental in producing fever.
"A reference to the meteorological tables will show that the atmosphere was
in general far from being moist; the dryness of the air indeed increased as we
advanced upwards, and it was remarkable at Egga, so that it can hardly be sup-
posed that the body was predisposed to disease by the atmosphere carrying off
its electricity by induction, or by impeding exhalation from the skin." (p. 176.)
Failing to trace the source of the disease in these and other chemical
or meteorological influences, the author falls back on the old etiology
of marsh miasma, or malaria, without attempting to explain what this is.
It is evident, however, that the conditions which have been generally
recognized as the most fertile sources of this poison, existed, in great
force, in the Niger.
"We had traversed (and slowly) a country of a character recognized as emi-
nently fertile in the production of fever. Swamps in a rich alluvial soil abounded.
The islands and banks were everywhere more or less inundated. The vegetation
was rank and profuse. The ships' companies were necessarily a good deal
exposed on deck during the day, and were sometimes harassed by frequent
anchoring and weighing in ascending the river. The actual labour cannot be
said to have been great, but there was a degree of restless anxiety inseparable
from being constantly liable to be called on deck. The upper deck was much
lumbered, in a degree preventing the free circulation of the air." (pp. 177-8.)
Dr. M'William shows that contagion had no share in producing the
disease. He also proves that one attack does not secure against a second.
In the section on treatment the author briefly details the result of his
own observation and experience. His practice seems to have been
judicious, but it presents no feature of novelty. In fact, we are much
disposed to believe that, as in the case of Dr. M'William's predecessors,
the course of the disease and its fatal issue were, in no way, checked by
the curative means employed. For these means we must refer to the
work itself. We fully agree with the author in the following obser-
vation :
"'Pessimum segroCoelum est, quod aegrum facit.' The most important step
in the treatment of African fever is comprehended in this maxim of Celsus. By
common admission of all who have served on the western coast, the causes of
fevers exist in a state of concentration in the rivers; and hence, as a general
rule, the greatest amount of mortality will be found on board those ships whose
crews are most employed in river service. I have no hesitation in saying that
in most instances, a favorable turn in the form of the fever, even in its earlier
stages, will attend a speedy removal from the locality where the disease ori-.
ginated ; and that after the fever has run its course, change of climate is indis-
pensable, if we wish to avoid intermittents, visceral complaints, and a host of
ailments that rarely fail to follow in its train." (p. 194.)
Our space will not allow us to notice any of the subjects treated of in
the last chapter. We cannot dismiss the work, however, without once
more recommending it to the notice not only of our own readers,?es-
pecially to all engaged in the public service,?but also of the general
reader : it is really interesting as a book of travels.

				

